# Fusion-inhibition peptide broadly inhibits influenza virus and SARS-CoV-2, including Delta and Omicron variants

**DOI:** 10.1080/22221751.2022.2051753

**Published:** 2022-03-30

**Authors:** Hanjun Zhao, Xinjie Meng, Zheng Peng, Hoiyan Lam, Chuyuan Zhang, Xinxin Zhou, Jasper Fuk-Woo Chan, Richard Yi Tsun Kao, Kelvin Kai-Wang To, Kwok-Yung Yuen

**Affiliations:** a State Key Laboratory of Emerging Infectious Diseases, Li Ka Shing Faculty of Medicine, The University of Hong Kong, Pokfulam, Hong Kong Special Administrative Region, People’s Republic of China; b Department of Microbiology, School of Clinical Medicine, Li Ka Shing Faculty of Medicine, The University of Hong Kong, Pokfulam, Hong Kong Special Administrative Region, People’s Republic of China; c Centre for Virology, Vaccinology and Therapeutics, Hong Kong Science and Technology Park, Hong Kong Special Administrative Region, People’s Republic of China; d Carol Yu Centre for Infection, Li Ka Shing Faculty of Medicine, The University of Hong Kong, Pokfulam, Hong Kong Special Administrative Region, People’s Republic of China; e Guangzhou Laboratory, Guangzhou Province, China

**Keywords:** Antiviral peptide, Delta variant, fusion, influenza virus, Omicron variant, SARS-CoV-2

## Abstract

Pandemic influenza virus and SARS-CoV-2 vaiants have posed major global threats to public health. Broad-spectrum antivirals blocking viral entry can be an effective strategy for combating these viruses. Here, we demonstrate a frog-defensin-derived basic peptide (FBP), which broadly inhibits the influenza virus by binding to haemagglutinin so as to block low pH-induced HA-mediated fusion and antagonizes endosomal acidification to inhibit the influenza virus. Moreover, FBP can bind to the SARS-CoV-2 spike to block spike-mediated cell–cell fusion in 293T/ACE2 cells endocytosis. Omicron spike shows a weak cell–cell fusion mediated by TMPRSS2 in Calu3 cells, making the Omicron variant sensitive to endosomal inhibitors. In vivo studies show that FBP broadly inhibits the A(H1N1)pdm09 virus in mice and SARS-CoV-2 (HKU001a and Delta)in hamsters. Notably, FBP shows significant inhibition of Omicron variant replication even though it has a high number of mutations in spike. In conclusion, these results suggest that virus-targeting FBP with a high barrier to drug resistance can be an effective entry-fusion inhibitor against influenza virus and SARS-CoV-2 in vivo.

## Introduction

Influenza viruses that caused pandemic and seasonal outbreaks have repeatedly overwhelmed healthcare institutions and affected socioeconomic activities. The suboptimal effectiveness of currently available anti-influenza drugs against certain strains was evidenced by the high mortality rates (>30%) of Influenza A(H5N1) and A(H7N9) virus-infected patients [1,2]. Drug-resistant viruses can emerge quickly in patients while on treatment with specific anti-influenza drugs, such as oseltamivir and baloxavir [3,4]. Moreover, resistant viruses against anti-influenza-neutralizing monoclonal antibodies could be identified after extensive virus passaging [5,6]. Besides the on-going influenza outbreaks, the pandemic SARS-CoV-2 (COVID-19) has significantly affected the globle for more than 2 years at the writing time. SARS-CoV-2 variants and circulating seasonal influenza virus may co-infect with increased severity during the influenza season [7–9]. These circulating influenza virus and SARS-CoV-2 variants reveal our poor capability in responding to the threats of emerging or re-emerging viruses with the currently available antivirals [1,2]. Thus, broad-spectrum agents inhibiting influenza virus and SARS-CoV-2 with a low possibility to induce drug resistance are urgently needed for combating the emergence of novel viruses.

Antiviral peptides with broad-spectrum antiviral activities against influenza virus and/or coronavirus have shown promising prospects with little metabolic toxicity [10–16]. Due to the lack of proofreading of RNA polymerases in RNA viruses, SARS-CoV-2 variants were not infrequently found in patients during the galloping pandemic [17]. Similarly, drug-resistant influenza virus mutants emerged during treatment by specific antivirals, especially the small molecular compounds, including neuraminidase inhibitors, M2 and polymerase inhibitors [3,4]. Naturally existing in almost all multicellular plants and animals, defensins have broad antiviral activities against influenza virus, coronavirus and other viruses [18,19]. Moreover, defensin-induced resistant viruses have not been reported, which are consistent with our finding that defensin-derived peptide P9R did not induce drug-resistant virus even after extensive virus passaging in P9R [13]. Chloroquine with broad-spectrum antiviral activities targeting host factors was effective in inhibiting pH-dependent viruses in vitro and in vivo [20,21], but not effective in vivo in some other studies with unclear reasons [22–24]. Camostat, the host-targeting inhibitor of TMPRSS2, has been shown to inhibit SARS-CoV, SARS-CoV-2 and other viruses [25–27]. The broad-spectrum antiviral activity and low chance of inducing drug-resistant viruses make the defensin-derived peptides and host-targeting antivirals promising drug development candidates.

This study demonstrated a short frog-defensin-derived basic peptide (FBP), which could broadly inhibit influenza A/B virus and SARS-CoV-2 variants. Mechanistic studies showed that FBP could block the low-pH-induced HA-mediated fusion of A(H1N1), A(H7N7) and FluB viruses. FBP could also inhibit endosomal acidification from suppressing the fusion of influenza virus and SARS-CoV-2 by the endocytic pathway. Further in vivo studies showed that FBP could significantly inhibit A(H1N1)pdm09 virus in mice and SARS-CoV-2 replication in hamsters. Interestingly, FBP could potently inhibit the Omicron variant, which had many mutations in spike and was more sensitive to endosomal inhibitors. Overall, we demonstrated that endosomal fusion inhibitor FBP with dual antiviral functions by directly targeting influenza HA to block HA conformational change and inhibiting endosomal acidification from suppressing pH-dependent viruses (i.e. blocking influenza virus and SARS-CoV-2 fusion in endolysosomes) could broadly inhibit influenza virus and SARS-CoV-2 replication in vivo.

## Results

### Basic peptide FBP broadly inhibited influenza virus

**Figure 1.**
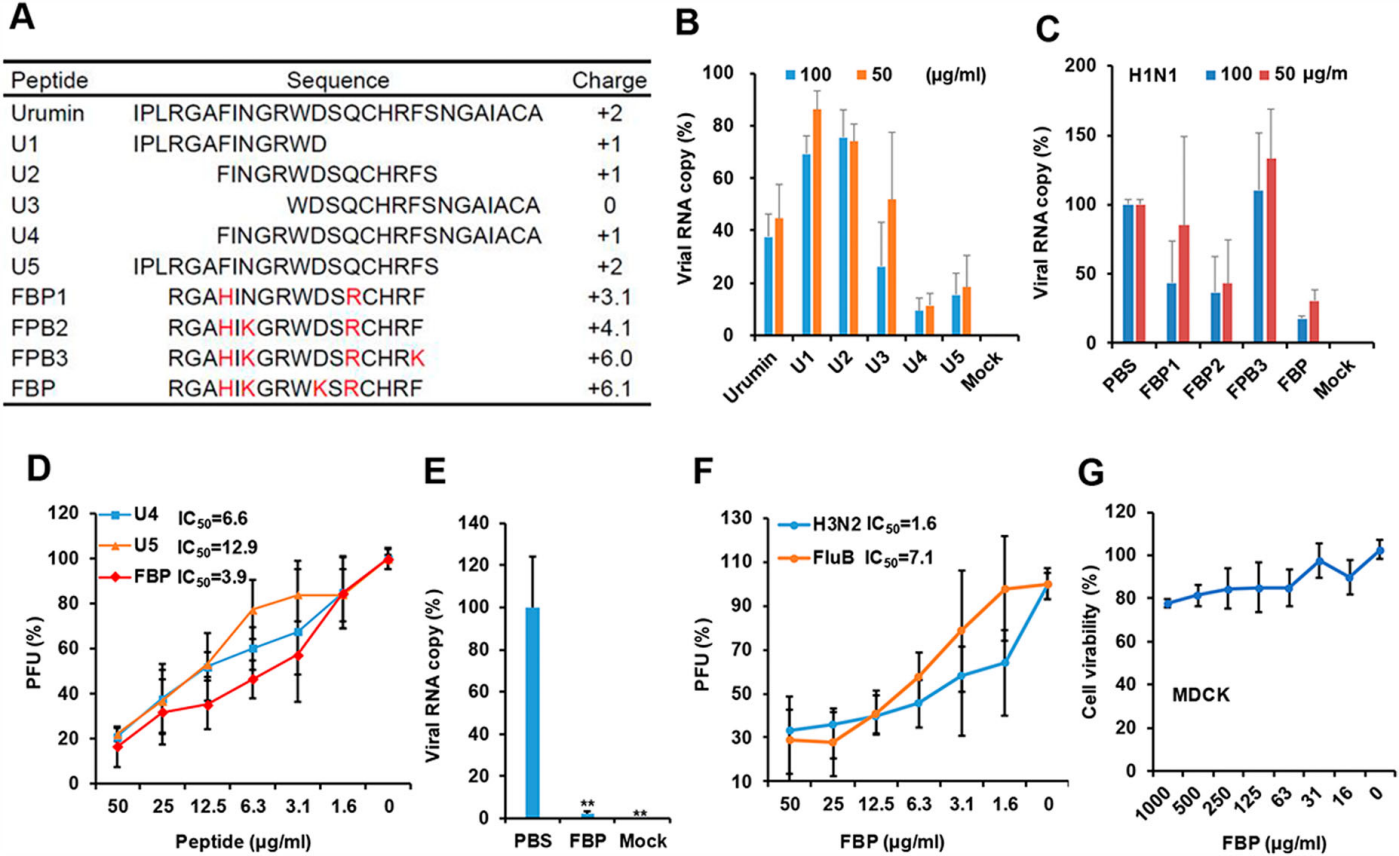
Short basic peptides inhibited influenza A and B viruses. (A) Peptide sequences and positive charges calculated by PepCalc of InnovaGen related to pH 7.0. (B) The antiviral activity of peptides against the A(H1N1) virus (*n* = 3). (C) Frog-defensin-derived basic peptides (FBP) inhibited the A(H1N1) virus (*n* = 3). The virus was treated with peptides by the indicated concentrations for infection in MDCK cells. Viral RNA copies in cell lysates were measured by RT-qPCR at 5 hpi. Viral RNA copy (%) was defined as the percentage of RNA copies of treated samples relative to those of untreated viruses. (D) Antiviral activity of peptides against the A(H1N1) virus was measured by the plaque reduction assay (*n* = 3). (E) Antiviral activity of FBP (50 μg ml^−1^) against A(H1N1) virus was measured by RT-qPCR to test the viral RNA copies in supernatants of 18 hpi (n = 3). ** indicates *P* < .01. *P*-value was calculated by the two-tailed Student’s *t*-test compared with PBS. (F) Antiviral viral activities of FBP against A(H3N2) and influenza B (FluB) viruses were measured by the plaque reduction assay (*n* = 4). (G) Cytotoxicity of FBP in MDCK cells (*n* = 3). Data are presented as mean ± SD of indicated biological samples.

Our previous studies showed that basic antiviral peptides from mouse beta-defensin could broadly inhibit respiratory viruses [12,13]. Here, we aimed to investigate whether a virus-binding peptide from frog defensin could be modified with a more positive charge to acquire dual-functional activities against the virus by the direct binding and inhibition of endosomal acidification to enhance the antiviral activity. We designed short peptides from a frog defensin Urumin which could bind to the HA stem of group 1 influenza A virus [10], and we found that U4 and U5 showed more potent antiviral activity than that of Urumin against A(H1N1) virus (Figure 1(A,B)). To find more potent antiviral peptides, we designed four peptides (FBP, FBP1, FBP2, FBP3) with a more positive charge (Figure 1(A)). We demonstrated that a short peptide (FBP) with a positive charge (+6.1) showed better antiviral activity than other basic peptides (Figure 1(C)). Moreover, FBP could significantly inhibit the A(H1N1) virus (IC_50_ = 3.9 μg ml^−1^, Figure 1(D)). The IC_50_ was lower than the IC_50_ of U4 (6.6 μg ml^−1^) and U5 (12.9 μg ml^−1^). The anti-H1N1 activity of FBP was further confirmed by the inhibition of viral multicycle growth (Figure 1(E)), which showed that FBP could significantly inhibit 44-fold viral replication. More interestingly, FBP could also inhibit A(H3N2) (IC_50_ = 1.6 μg ml^−1^) and FluB (IC_50_ = 7.1 μg ml^−1^) viruses (Figure 1(F)). The cytotoxicity analysis showed that no significant cytotoxicity was detected in MDCK cells treated with 1 mg ml^−1^ of FBP (TC_50_ > 1 mg ml^−1^, Figure 1(G)). No significant haemolysis was observed when Turkey red blood cells (RBC) were treated with FBP (Figure S1). These results indicated that the short basic peptide FBP could potently inhibit influenza A (group 1 and group 2) and Flu B viruses without an obvious cytotoxic effect on host cells.

### FBP blocked HA-mediated fusion and endosomal acidification

**Figure 2.**
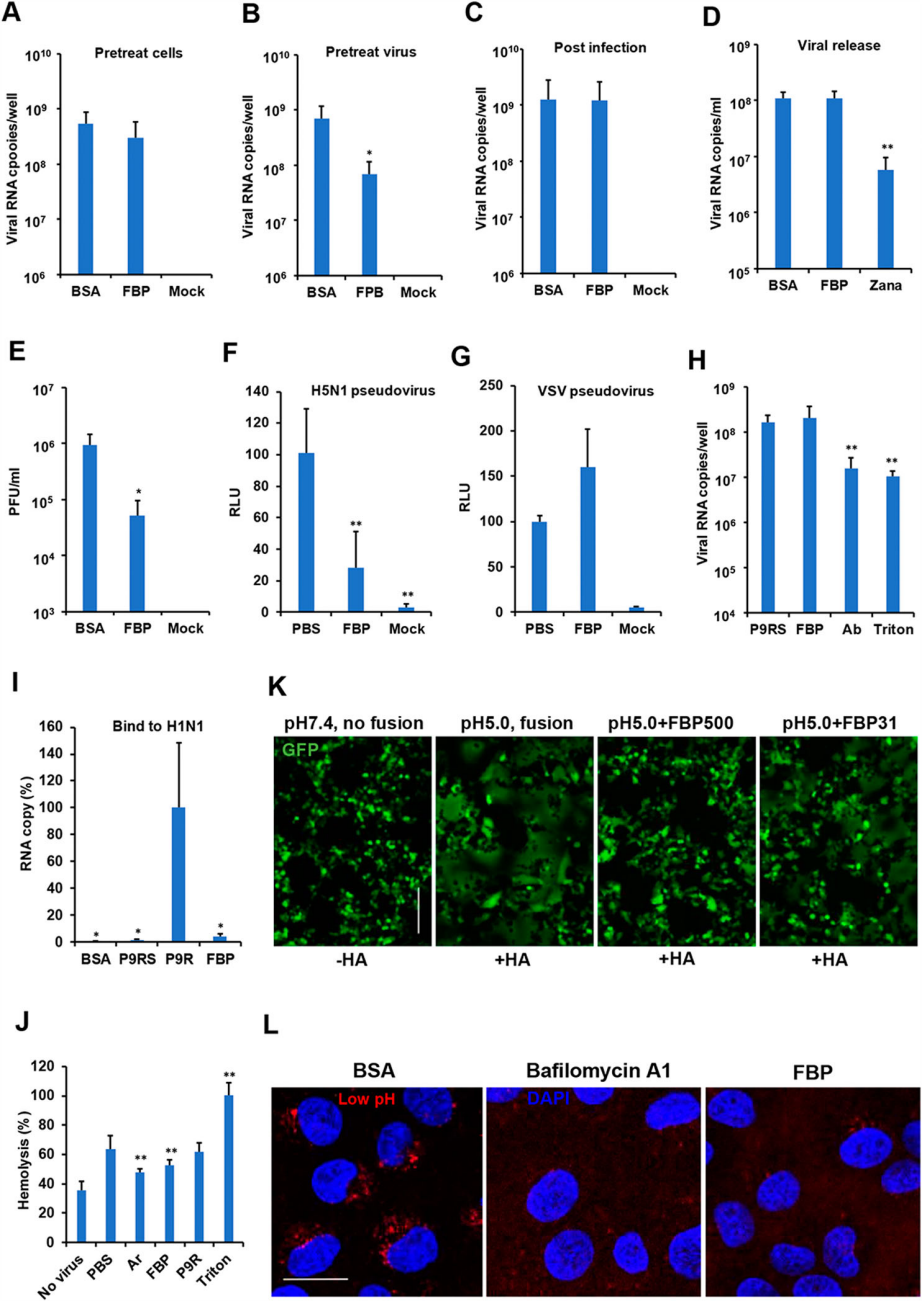
FBP blocked HA-mediated cell–cell fusion and endosomal acidification. (A) FBP did not inhibit the virus when cells were treated before viral infection (Pretreat cells, *n* = 5). (B) FBP inhibited viral replication when the virus was treated with FBP (25 μg ml^−1^) before viral infection (Pretreat virus, *n* = 5). (C) FBP did not inhibit the virus when cells were treated after viral infection (Post-infection, *n* = 5). Viral RNA copies in cell lysates were measured by RT-qPCR at 5 hpi. (D) FBP did not inhibit viral release. Viral RNA copies in supernatants were measured by RT-qPCR at 8 hpi. (E) FBP (50 μg ml^−1^) inhibited A(H1N1) by targeting the virus (*n* = 3). *P* values were calculated by comparison with BSA. (F–G) FBP inhibited H5N1-pseudovirus entry (*n* = 5), but not VSV entry. H5N1 or VSV pseudovirus was treated with FBP (50 μg ml^−1^) for cell entry. Luciferase expression was measured at 24 hpi. Untreated pseudovirus (PBS) and uninfected cells (Mock) were served as controls. *P-*values were calculated by comparison with PBS. (H) FBP (25 μg ml^−1^) did not affect A(H1N1) virus attachment (*n* = 3). The A(H1N1) virus was treated with FBP, P9RS (peptide without antiviral activity), neutralizing antibody (Ab) and Triton X-100 for attachment to MDCK cells at 4°C. The attached virus was measured by RT-qPCR. *P*-values were calculated by comparison with P9RS. (I) FBP could not capture A(H1N1) viral particles (*n* = 3). FBP (2 μg), P9R (positive control), P9RS and BSA were coated on an ELISA plate. The A(H1N1) virus was added to the ELISA plate for binding. Viral RNA copies were measured to show the bound virus. *P-*values were calculated by comparison with P9R. (J) FBP (50 μg ml^−1^) blocked RBC haemolysis induced by A(H1N1) virus at low pH 5.0 (*n* = 8). * indicates *P* < .05. ** indicates *P* < .01. *P-*values were calculated by the two-tailed Student’s *t*-test compared with PBS. Data are presented as mean ± SD of independent biological samples. (K) FBP inhibited HA-mediated cell–cell fusion triggered by low pH5.0. HA of the A(H7N7) virus and GFP were expressed in 293T cells. Cells treated by FBP (500 and 31 μg ml^−1^) or untreated cells were challenged by pH 5.0. Cells challenged by pH 7.4 were served as no-fusion control. Scale bar = 100 μm. Experiments were repeated twice. (L) FBP inhibited endosomal acidification. MDCK cells were treated with BSA (25 μg ml^−1^), FBP (25 μg ml^−1^) or bafilomycin A1 (50 nM) and pH-sensitive dye. Scale bar = 20 μm. Experiments were repeated twice.

To investigate the antiviral mechanism of FBP against influenza virus, cells were treated with FBP before A(H1N1) virus infection, but no antiviral activity was detected (Figure 2(A)). When the virus was treated with FBP before infection, FBP could significantly inhibit viral replication (Figure 2(B)). When infected cells were treated with FBP after viral infection, FBP did not inhibit viral replication in cells (Figure 2(C)) and viral release in supernatants (Figure 2(D)). However, FBP did not inhibit rhinovirus replication when cells or viruses were treated with FBP before or after viral infection (Figure S2). These results indicated that the antiviral activity of FBP might mainly rely on targeting A(H1N1) virus before viral entry and not rely on non-specific targeting host. To further confirm the mechanism, we treated the A(H1N1) virus (1 × 10^6^ PFU ml^−1^) with FBP (500 μg ml^−1^) and then diluted the FBP-treated virus by 10, 000 folds for the plaque assay. After the 10,000-fold dilution, FBP at the concentration of 0.05 μg ml^−1^ (less than the IC_50_ of 3.9 μg ml^−1^) could still significantly reduce the plaque number (Figure 2(E)), which further confirmed that the antiviral activity of FBP mainly relied on targeting virus and was not affected by the extreme dilution. To evaluate if FBP could disrupt viral particles, we showed that intact A(H1N1) virus treated with FBP, similar to the untreated virus, could be detected by transmission electron microscopy (TEM). However, the intact viral particle was not detected when the virus was treated with Triton X-100 (Figure S3). These results indicated that the antiviral activity of FBP mainly relied on directly targeting the virus, which interfered with viral infection at the early stage without disrupting the viral particles.

Next, we used A(H5N1) pseudovirus, which only expressed HA and NA proteins, to test whether FBP could affect pseudovirus entry. As shown in Figure 2(F), FBP could significantly inhibit the entry of A(H5N1) pseudovirus but not inhibit VSV pseudovirus entry (Figure 2(G)), which indicated that FBP most likely interfered with the early step of viral infection by targeting HA. FBP binding to HA was further confirmed by HA pull-down assay (Figure S4), while FBP did not reduce the viral attachment (Figure 2(H)), which also suggested that FBP did not disrupt viral particles because the viral RNA copies of the attached virus were significantly reduced when the virus was disrupted by Triton X-100 (Figure 2(H)). We further confirmed that FBP did not have haemagglutination inhibition (HAI) activity against A(H1N1) compared with neutralization antibody (Figure S5). Furthermore, unlike P9R, which is bound to viral surface HA and captures viral particles [13], FBP could not capture viral particles, as shown in the capture assay (Figure 2(I) and Figure S6), which implicated that FBP was less likely to bind to the head region of HA. Considering the broad-spectrum antiviral activities of FBP against group 1 and 2 influenza A virus and FluB virus, we hypothesized that FBP might bind to the HA stem region to interfere with HA conformational change. Consistently, we demonstrated that FBP could significantly inhibit RBC haemolysis induced by group 1 A(H1N1) virus at pH 5.0 condition (Figure 2(J)). Furthermore, it was confirmed that FBP could block group 2 A(H7N7)-HA mediated cell fusion triggered by the low pH in 293T cells (Figure 2(K)) and FluB virus-mediated cell fusion in MDCK cells (Figure S7). FBP could inhibit the pH5.0 induced cell-fusion sizes (10–20 μm, normal sizes of unfused cells), which were smaller than fusion cells (sizes >50 μm) treated with pH5.0 only (Figure 2(K)). These results suggested that FBP might bind to the stem region to block the low pH-induced HA conformational change [28]. Finally, it was demonstrated that basic FBP could inhibit endosomal acidification, similar to the effect of bafilomycin A1 [13] in live cells (Figure 2(L)). These results indicated that FBP could have dual functions: blocked HA-mediated fusion by binding and inhibited endosomal acidification from interfering viral entry by the endocytic pathway.

### FBP inhibited SARS-CoV-2 by interfering with endosomal pH

**Figure 3.**
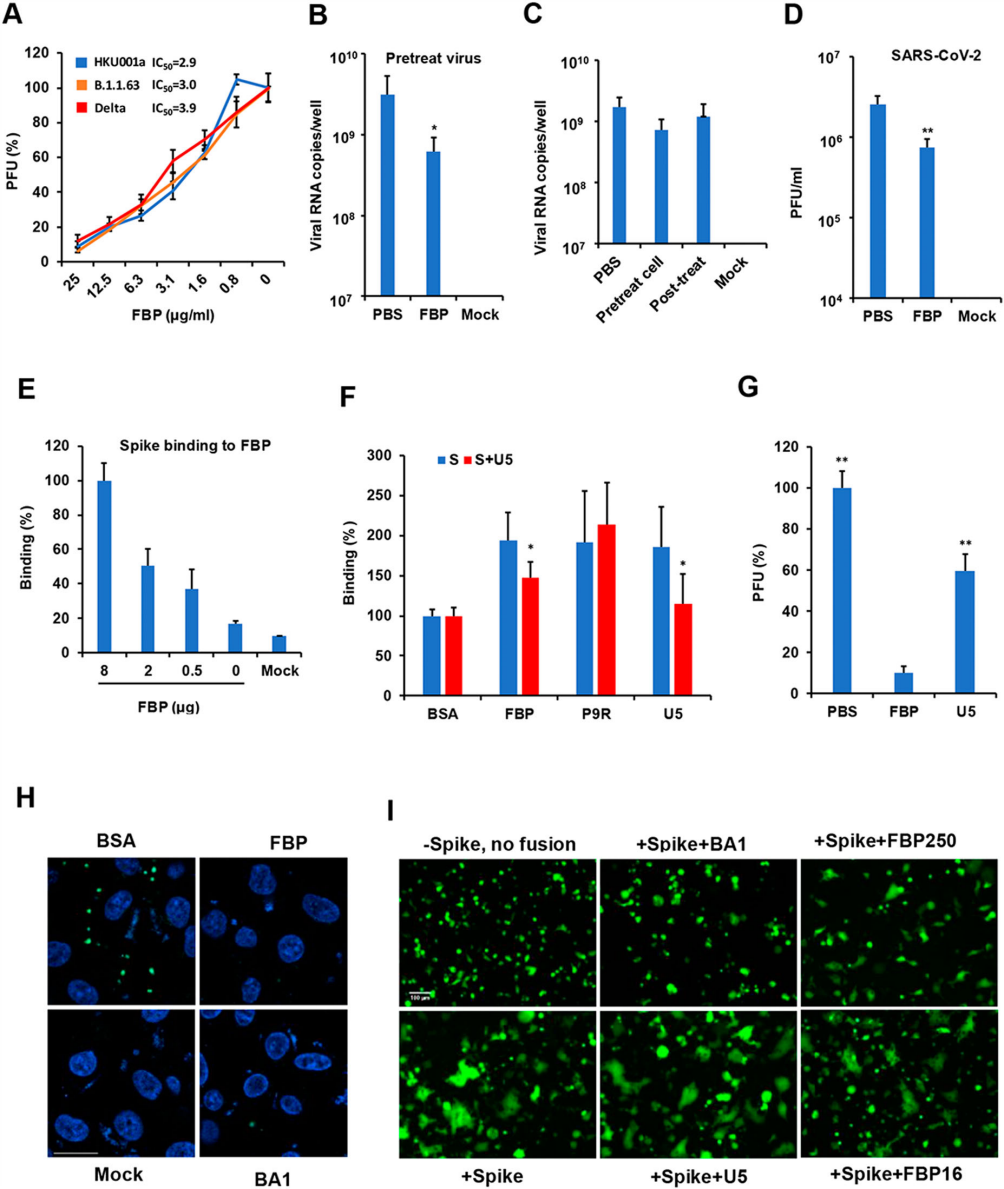
FBP inhibited SARS-CoV-2 infection in vitro. (A) Antiviral activity of FBP against SARS-CoV-2 in Vero-E6 cells (*n* = 3). SARS-CoV-2 variants were treated with FBP for cell infection. PFU (%) was normalized to the untreated viruses. (B) SARS-CoV-2 was treated with FBP (25 μg ml^−1^) and then infected cells for 1 h. Viral RNA copies in cell lysate were measured by RT-qPCR at 8 hpi (*n* = 5). (C) Cells were treated with FBP (25 μg ml^−1^) for 1 h before viral infection (Pretreat cell), and FBP was added to cells at 1 hpi (Post-treat). Viral RNA copies in cell lysate were measured by RT-qPCR at 8 hpi (*n* = 3). Mock, uninfected cells. (D) FBP inhibited SARS-CoV-2 by targeting the virus (*n* = 3). The virus (1 × 10^6^ PFU/ml) was treated by FBP (500 μg ml^−1^) and then was diluted to 1000 folds for the plaque assay. * indicates *P* < .05. *P*-values were calculated by the two-tailed Student’s *t*-test compared with PBS. (E) Dose-dependent binding of FBP to spike protein (*n* = 4). Spike binding to indicated FBP (8, 2 and 0.5 μg) and BSA (Mock) on the ELISA plate. Relative binding (%) was the OD values normalized to the OD value of FBP (8 μg). (F) FBP could bind to S protein, and U5 blocked the binding between FBP and S protein (*n* = 4). S protein of SARS-CoV-2 and S treated with U5 (S + U5) were added to the ELISA plate for binding to peptides coated on the ELISA plate. * indicates *P* < .05 compared with untreated S. (G) U5 (25 μg ml^−1^) showed weaker antiviral activity than that of FBP (25 μg ml^−1^) against SARS-CoV-2 (*n* = 4). The antiviral activity was measured by the plaque reduction assay. (H) FBP inhibited endosomal acidification. Vero-E6 cells were treated by BSA, FBP (25 μg/ml) or bafilomycin A1 (BA1, 25nM). The low pH indicator (green) showed the low pH in endosomes. Nuclei were stained by DAPI (blue). Untreated cells (Mock) were the negative control. Scale bar = 20 μm. Experiments were repeated twice. (I) FBP inhibited spike-ACE2-mediated cell–cell fusion. Co-cultured cells (293T/spike and 293T/ACE2 cells) were treated with FBP (250 and 16 μg ml^−1^), U5 (250 μg ml^−1^) and bafilomycin A1(BA1, 50 nM). Cells without spike (-spike) were served as the no-fusion control. Scale bar = 100 μm. Experiments were repeated twice.

To test the broad-spectrum antiviral activity of FBP, we demonstrated that FBP could inhibit variant SARS-CoV-2 infection, as revealed by the plaque reduction assay (Figure 3(A)). FBP could significantly inhibit viral replication when the virus was treated before infection (Figure 3(B)) but dot inhibited the virus when cells were treated before or after infection (Figure 3(C)). FBP did not significantly inhibit viral attachment, viral release (Figure S8) and the spike-ACE2 binding (Figure S9). We further demonstrated that FBP (50 μg ml^−1^) significantly inhibited SARS-CoV-2 even when the FBP-treated virus was 1000-fold diluted to let FBP drop to 0.5 μg ml^−1^ (Figure 3(D)). These results indicated that FBP mainly targeted viruses to inhibit viral infection. Next, we demonstrated that FBP and U5 could bind to spike protein (Figure 3(E)) and U5 could block the binding between FBP and spike (Figure 3(F) and Figure S10). However, U5 showed significantly weaker antiviral activity against SARS-CoV-2 infection than FBP (Figure 3(G)). These results suggested that the binding of U5 to spike protein itself could not inhibit SARS-CoV-2. We further demonstrated that FBP and bafilomycin A1 could inhibit endosomal acidification in Vero-E6 cells (Figure 3(H)) and inhibit spike-ACE2-mediated cell–cell fusion in 293T cells (Figure 3(I)). FBP and BA1 could inhibit the cell sizes of spike-ACE2-mediated cell–cell fusion (unfused cell sizes: 10–20 μm), which were smaller than the sizes (>50 μm) of fusion cells treated by U5 (+Spike + U5) or mock (+Spike). U5 did not show the inhibition of cell fusion. These results indicated that the notable antiviral activity and fusion inhibition activity of FBP on SARS-CoV-2 could be attributed to the inhibition of FBP on endosomal acidification, similar to the fusion inhibition of bafilomycin A1. We further demonstrated that FBP could not significantly inhibit SARS-CoV-2 replication in Calu-3 cells, in which SARS-CoV-2 relied on TMPRSS2 but not on endocytosis for viral infection (Figure S11), which was consistent with the activity of FBP against SARS-CoV-2 by inhibiting endosomal acidification.

### FBP broadly inhibited influenza the virus and SARS-CoV-2 in vivo

**Figure 4.**
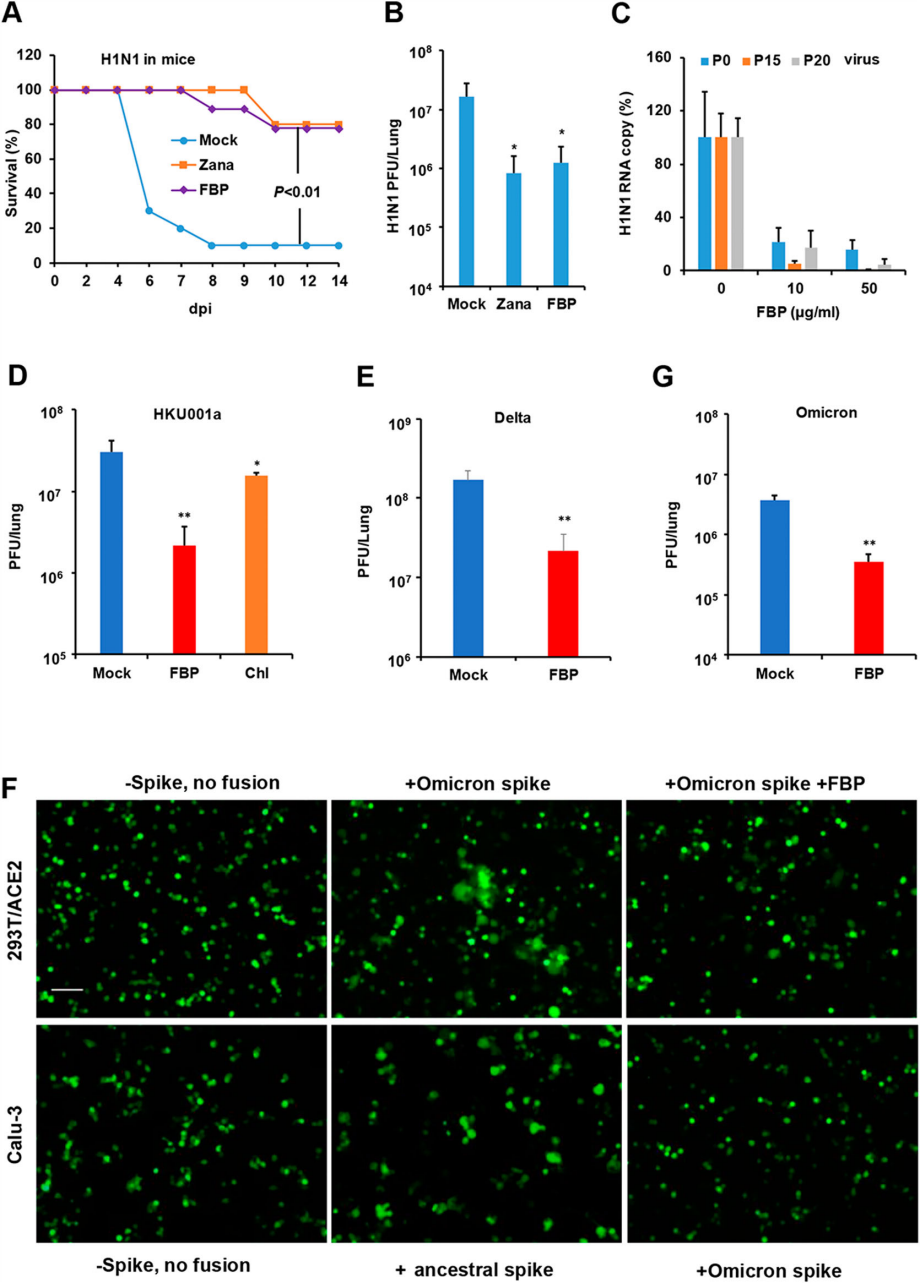
FBP inhibited influenza virus and SARS-CoV-2 in animals. (A) The survivals of A(H1N1)-infected mice intranasally treated with FBP (2 mg kg^−1^, *n* = 9), zanamivir (Zana, 1.6 mg kg^−1^, *n* = 5) or PBS (Mock, *n* = 9) at 6 hpi with two more doses the following day. *P-*value was calculated by the Gehan–Breslow–Wilcoxon test. (B) FBP inhibited A(H1N1) virus replication in mouse lungs (*n* = 5) at 2 dpi. (C) Antiviral efficiency of FBP against parent H1N1 (P0), 15-passaged virus (P15) and 20-passaged virus (P20) in MDCK cells (*n* = 3). Viral RNA copies in cell supernatants were measured at 18 hpi and normalized to the untreated virus (0). (D) FBP or chloroquine (Chl, 2 mg kg^−1^) inhibited SARS-CoV-2 (HKU001a) replication in hamster lungs at 2 dpi (*n* = 4). (E) SARS-CoV-2 (Delta, B.1.617.2) replication in hamster lungs treated with inhaled PBS (Mock, *n* = 8) or FBP (2 mg kg^−1^, *n* = 4). Antivirals were intranasally inoculated to animals at 8 hpi, and two more doses were given to hamsters the following day. (F) FBP effectively inhibited Omicron-spike-mediated fusion in 293T/ACE2 cells (upper panel) and Omicron-spike-mediated fusion in Calu-3 cells was not observed (lower panel). 293T/ACE2 cells or Calu-3 cells were co-cultured with 293T expressed with an ancestral spike from HKU001a or Omicron spike with or without the treatment of FBP (250 μg ml^−1^). 293T without spike (-spike) was used as the no fusion control. Scale bar = 100 μm. Experiments were repeated twice. (G) SARS-CoV-2 (Omicron, B.1.1.529) replication in hamster lungs treated with PBS (Mock, *n* = 4) or FBP (2 mg kg^−1^, *n* = 4). Infected hamsters were intranasally inoculated with PBS or FBP at 8 hpi, and two more doses were given to hamsters the following day. * indicates *P* < .05 and ** indicates *P* < .01 compared with mock. *P-*values were calculated by the two-tailed Student’s *t*-test. Data are presented as mean ± SD of independent biological samples.

To evaluate the antiviral efficacy of FBP in vivo, we challenged mice with the A(H1N1)pdm09 virus. Inhaled FBP could significantly increase the survival of challenged mice by 80% (Figure 4(A)) and inhibit viral replication in the lungs (Figure 4(B)). The protection conferred by FBP on infected mice was similar to that of zanamivir (Figure 4(A,B)). Considering the drug-resistant problems of anti-influenza drugs, we tested whether the drug-resistant mutants of the A(H1N1) virus could emerge when the A(H1N1) virus was cultured in the presence of FBP (Figure 4(C)). FBP could efficiently inhibit the replication of the passaged viruses (P15 and P20), with a similar efficiency against the virus without passage (P0).

To investigate the activity of FBP against SARS-CoV-2 in vivo, hamsters infected with ancestral SARS-CoV-2 (HKU001a) were intranasally inoculated with FBP to hamsters at 8 hpi. Two more doses were given to hamsters the following day. Because SARS-CoV-2 could reach peak viral titres in hamster lungs at 2 dpi [24], viral loads in hamster lungs were measured at 2 dpi. We demonstrated that FBP could significantly inhibit SARS-CoV-2 (HKU001a) replication in hamster lungs (Figure 4(D)). The endosomal inhibitor chloroquine against SARS-CoV-2 in vitro [29] could only show modest inhibition (2.1-fold) on SARS-CoV-2 replication in hamsters when administrated by intranasal inoculation. To confirm the broad antiviral activity of FBP on SARS-CoV-2 variants, we demonstrated that FBP could significantly inhibit Delta variant replication in hamster lungs (Figure 4(E)). Moreover, we showed that FBP could effectively inhibit Omicron-spike-mediated cell–cell fusion in 293T/ACE2 cells (Figure 4(F)). The omicron spike showed less cell–cell fusion in Calu-3 cells than the ancestral spike (Figure 4(F)), which was consistent with that the Omicron variant was sensitive to bafilomycin A1 and largely depending on the endocytic pathway but not on TMPRSS2 for viral replication [30]. Also, FBP could significantly inhibit Omicron variant (B.1.1.529) replication in hamsters, which demonstrated that the broad antiviral activity of FBS was not affected by the additional mutations in the Omicron spike (Figure 4(G)). These results suggested that endosomal fusion inhibitor FBP could significantly inhibit influenza virus and SARS-CoV-2 variants with high number mutations, which indicated the broad-spectrum activity of FBP against multiple viruses with a high barrier to drug resistance.

## Discussion

In this study, we provided a proof of concept that a virus-targeting peptide could be developed with dual antiviral functions by targeting virus and host endocytosis. We demonstrated that the fusion-inhibition peptide FBP could broadly inhibit influenza virus and SARS-CoV-2 by interfering the viral fusion by the endocytic pathway and showed potently antiviral activity against the influenza virus in mice and SARS-CoV-2 variants in hamsters, suggesting that an endosomal fusion inhibitor blocking viral entry can be an effective strategy to show broadly antiviral activity in vivo.

Peptides binding to the HA stem region have inhibited group 1 influenza A viruses [10,28]. However, the stem region is not conserved in the influenza virus [31], which may challenge finding a universal antiviral against the influenza virus by targeting the HA stem region. Here, we reported that FBP can inhibit HA-mediated cell–cell fusion of group 1 A(H1N1), group 2 A(H7N7) and FluB viruses and provided the evidence that a basic peptide could be a fusion inhibitor with broad-spectrum activities against influenza A and B viruses. Moreover, basic FBP could bind to viral surface proteins, such as HA and spike and inhibit the influenza virus and SARS-CoV-2 by preventing the HA- or spike-mediated cell–cell fusion by the endocytic pathway. The exact mode of broad binding activity of FBP to HA or spike, which might be due to the structure-charge-based binding and the flexible structure of peptide for better fitting different binding pockets, warrants further co-binding studies for confirmation. Broad-spectrum antiviral chloroquine, which elevates the endosomal pH of host cells without binding to the virus, had inhibited different pH-dependent viruses in vitro and in vivo [20,21]. Several studies demonstrated the lack of antiviral activity of chloroquine in animal studies and clinical trials when the drug was administrated by oral or IP injection [23,24,32–34]. These treatment failures might be due to the low plasma concentration of chloroquine (<0.5 μg ml^−1^), lower than the IC_50_ (1–5 μg ml^−1^) of chloroquine against SARS-CoV-2 [23,24]. Here, we demonstrated the endosomal fusion inhibitor (virus-targeting FBP alone) could broadly inhibit A(H1N1) virus and SARS-CoV-2 replication in vivo when administrated through intranasal inhalation. However, the inhaled host-targeting chloroquine alone could only show modest antiviral activity in vivo. This might indicate that the virus-targeting FBP, binding to viral proteins, could more effectively inhibit pH-dependent virus replication in vivo compared with the host-targeting chloroquine although both of them could inhibit the endocytic pathway of viral infection.

Because of the high mutation rates resulting in antigenic drift, seasonal influenza vaccines could only provide 10–60% of protection during the past decades. Since the discovery of neuraminidase inhibitors in 1990 [35], M2 inhibitor [36] and polymerase inhibitor [4] have been found. However, viruses resistant to these inhibitors quickly emerged even during the clinical trials [3,4,37]. In addition, with the circulation of pandemic SARS-CoV-2, different variants have been reported. Thus, antivirals with new antiviral mechanisms and broad-spectrum activities against viruses with a high barrier to drug resistance are needed for treating influenza virus and coronavirus. FBP could broadly inhibit influenza virus and SARS-CoV-2 variants and no drug-resistant influenza virus was found after 20 viral passages in the presence of FBP, which suggests the high barrier to drug resistance of FBP.

Currently, the rapid RT-PCR test can identify patients in the early period of infection, which allows drug atomization for the early treatment. For late presenters, when the peak of the viral load has passed, antiviral drugs given by any routes are difficult to improve the outcome. Immunomodulatory agents, such as steroids, may play a more important therapeutic role in the late cases of SARS-CoV-2 infection. Recently, the inhaled neuraminidase DAS181 has shown promising results for treating parainfluenza viral infection in phase 2 [38] and 3 clinical trials (NCT04298060), and inhaled vaccines were effective activity against SARS-CoV-2 [39,40], which support the use of inhalational drugs for treating respiratory viral diseases. The topical administration of drugs by atomization inhalation in the early period of infection may not only enhance the antiviral efficacy in the lungs but also reduce the potential side effects compared with systemic administration by oral or intravenous injection. Our inhaled treatment results suggest that the peptidic fusion inhibitor can be a broad-spectrum antiviral candidate for treating influenza virus and SARS-CoV-2 infection.

## Material and methods

### Cell and virus culture

Madin Darby canine kidney (MDCK, CCL-34), Vero-E6 (CRL-1586), Vero-E6-TMPRSS2, 293T (CRL-3216), and Calu-3 (HTB-55) cells obtained from ATCC (Manassas, VA, USA) were cultured in Dulbecco minimal essential medium (DMEM) or MEM supplemented with 10% fetal bovine serum (FBS), 100 IU ml^−1^ penicillin and 100 μg ml^−1^ streptomycin. The virus strains used in this study included A/Hong Kong/415742M/2009 (H1N1), A/Hong Kong/4801/2014 (H3N2), A/Netherlands/219/2003 (H7N7) [41], A/Anhui/1/2013 (H7N9), influenza B/TW/70555/05 (FluB), SARS-CoV [12], SARS-CoV-2 (HKU001a) with S1/S2 multi-basic site deletion, SARS-CoV-2 (B.1.1.63, D614G), SARS-CoV-2 (B.1.617.2) and SARS-CoV-2 (B.1.1.529) [30,42,43].

### Plaque reduction assay

Peptides were synthesized by ChinaPeptide. The antiviral activity of peptides was measured using a plaque reduction assay. Briefly, peptides or bovine serum albumin (BSA, 0.2–50.0 μg ml^−1^) was premixed with 50 PFU of virus in PBS at room temperature. After 45–60 min of incubation at room temperature, the peptide-virus mixture was transferred to MDCK or Vero-E6 cells, correspondingly. At 1 h post-infection, infectious media were removed, and 1% low melting agar was added to cells. Cells were fixed using 4% formalin at 2–3 day post-infection. Crystal violet (0.1%) was added for staining, and the number of plaques was counted.

### Viral RNA extraction and RT-qPCR

Viral RNA was extracted by Viral RNA Mini Kit (QIAGEN, Cat^#^ 52906, USA), according to the manufacturer’s instructions. Extracted RNA was reverse-transcribed to cDNA using PrimeScript II 1st Strand cDNA synthesis Kit (Takara, Cat^#^ 6210A) with GeneAmp® PCR system 9700 (Applied Biosystems, USA). The cDNA was then amplified using specific primers (Table S1) for detecting the virus using LightCycle® 480 SYBR Green I Master (Roach, USA). For quantitation, 10-fold serial dilutions of standard plasmid equivalent to 10^1^–10^6^ copies per reaction were prepared to generate the calibration curve. Real-time qPCR experiments were performed using the LightCycler® 96 system (Roche, USA).

### Antiviral multicycle growth assay

Influenza viruses were treated with peptides and then infected MDCK cells (0.005 MOI). After 1 h infection, infectious media were removed, and fresh media with supplemental peptides (50 μg ml^−1^) were added to infected cells for virus culture. At 18 h post-infection, the supernatants of infected cells were collected for the RT-qPCR assay to determine the viral titres in cell supernatants.

### Cytotoxicity assay

Cytotoxicity of peptides was determined by detecting 50% cytotoxic concentration (CC_50_) using a tetrazolium-based colorimetric MTT assay. Briefly, MDCK cells were seeded in a 96-well cell culture plate at an initial density of 2 × 10^4^ cells per well in DMEM supplemented with 10% FBS and incubated overnight. Cell culture media were removed, and then DMEM supplemented with various concentrations of peptides and 1% FBS was added to each well. After 24 h incubation at 37°C, MTT solution (5 mg ml^−1^, 10 μl per well) was added to each well for incubation at 37°C for 4 h. Then, 100 μl of 10% SDS in 0.01M HCl was added to each well. After further incubation at room temperature with shaking overnight, the plates were read at OD_570_ using VictorTM X3 Multilabel Reader (PerkinElmer, USA). Cell culture wells without peptides were used as the experiment control, and medium only served as a blank control.

### Haemolysis and haemolysis inhibition assay

Serially diluted peptide FBP in PBS was incubated with turkey red blood cells for 1 h at 37°C. PBS was used as a 0% lysis control and 0.1% Triton X-100 as a 100% lysis control. Plates were centrifuged at 350 g for 3 min to pellet non-lysed red blood cells. Supernatants used to measure haemoglobin release were detected by absorbance at 450 nm. For the haemolysis inhibition assay, FBP (200 μg ml^−1^), P9R (200 μg ml^−1^) or arbidol (100 μg ml^−1^) were mixed with or without the same volume of H1N1 virus (HA titre > 128) for 1 h, and then 60 μl of 2% turkey red blood cells was added for 15 min. PBS and Triton X-100 (0.1%) were included as the negative and positive control of haemolysis. The precipitated erythrocytes were incubated with sodium citrate solution (pH of 4.9) for 25 min. The haemoglobin release in supernatants was detected at 450 nm.

### Transmission electron microscopy assay

To determine the effect of FBP on viral particles, the A(H1N1) virus was treated with 200 μg ml^−1^ of FBP, PBS, or Triton X-100 (0.15%) for 1 h. The virus was fixed by formalin overnight and then applied to continuous carbon grids. The grids were transferred into 4% uranyl acetate and incubated for 1 min. After removing the solution, the grids were air-dried at room temperature. For each sample, two to three biological samples were done for taking TEM images by FEI Tecnal G2-20 TEM.

### Pseudovirus assay

H5N1 pseudotype virus [44] bearing H5N1 HA and NA and Vesicular stomatitis virus (VSV) pseudotype virus were treated with PBS or FBP (50 μg ml^−1^) in PBS and then incubated at RT for 1 h. MDCK/293T cells were infected with the treated pseudotype virus for 1 h. Cells without pseudotype virus infection served as the baseline control of luciferase protein. After 18 h cell culture, cell lysates were collected, and the luciferase protein was measured by the Luciferase assay system (Promega) in a Victor X3 Multilabel reader (PerkinElmer). The luminescence reading was normalized to 1mg protein.

### Virus-induced cell fusion assay

MDCK cells were transfected with pGFP. Eight hours later, cells were infected with 1MOI of FluB virus. At 18 h post-infection, cells were treated with PBS or FBP (500 and 31 μg ml^−1^) for 1 h, and they were treated by pH 5.0 or pH 7.4 for 10 min. After removing the pH buffer, cells were cultured at 37°C for 4 h with complete media. Fusion pictures were taken at 4 h after pH treatment.

### HA mediated cell fusion assay

The 293T cells were co-transfected with pGFP and pH7N7-HA. At 24 h post-transfection, cells were treated with PBS or FBP (500 and 31 μg ml^−1^) or U5 (500 μg ml^−1^) for 1 h and then treated with pH 5.0 or pH 7.4 for 10 min. After removing the pH buffer, cells were cultured at 37°C for 4 h with complete media. Fusion pictures were taken at 4 h after pH treatment.

### Spike-ACE2-mediated cell fusion assay

The spike of SARS-CoV-2 (HKU001a and Omicron variant), pACE2-human, or pGFP was transfected to 293T cells for protein expression. After 24 h, to trigger the spike-ACE2-mediated cell fusion, 293T-Spike-GFP cells were co-cultured with 293T-ACE2 or Calu-3 cells with or without the supplement of peptide (250 and 16 μg ml^−1^) or bafilomycin A1 (50 nM). The 293T-GFP cells were co-cultured with 293T-ACE2 or Calu-3 cells as the no-fusion negative control. After 6 h of co-culture, five fields were randomly selected in each well to take the cell fusion pictures by fluorescence microscopes.

### Peptide binding assay

Peptides (0.5–8.0 μg per well), including FBP, P9R, P9RS (13) or ACE2 (100 ng) dissolved in H_2_O, were coated onto ELISA plates and incubated at 4°C overnight. Then, 2% BSA was used to block plates at 4°C overnight. For virus or spike protein binding to peptides, viruses or spike protein were diluted in PBS and then were added to ELISA plates for binding to the coated peptides at room temperature for 1 h. After washing the unbound viruses or spike protein, the bound viruses were lysed by RLT buffer of RNeasy Mini Kit (Qiagen, Cat^#^ 74106) for viral RNA extraction. Viral RNA copies of binding viruses were measured by RT-qPCR. The bound spike protein was detected by anti-His-HRP (Invitrogen, Cat^#^ R93125, 1: 2,000) or rabbit anti-spike (Sino, Cat^#^ 40590-T62, 1:8000) with secondary goat-anti-rabbit HRP (Invitrogen, Cat^#^ 656120, 1:4000) by reading OD_450_.

### Endosomal acidification assay

Endosomal acidification was detected with a pH-sensitive dye (pHrodo Red dextran, Invitrogen, Cat^#^ P10361 and pHrodo Green zymosan, Invitrogen, Cat^#^ P35365), according to the manufacturer’s instructions, as previously described but with slight modification [3]. First, MDCK or Vero-E6 cells were treated with BSA (25.0 μg ml^−1^), FBP (25.0 μg ml^−1^) and bafilomycin A1 (50 nM) at 4°C for 15 min. Second, MDCK or Vero-E6 cells were added with 100 μg ml^−1^ of pH-sensitive dye and DAPI and then incubated at 4°C for 15 min. Before taking images, cells were further incubated at 37°C for 15 min and then they were washed twice with PBS. Finally, PBS was added to cells, and images were taken immediately with a confocal microscope (Carl Zeiss LSM 700, Germany).

### Rearing of virus-resistant mutant

The A(H1N1) virus was treated with FBP and then infected MDCK cells. After 1 h infection, MEM media with the supplement FBP (10 μg ml^−1^ for the initial 10 passages and 50 μg ml^−1^ for the passages from 11 to 20) were added to cells for virus culture. At 24 h post-infection, viruses were collected for the next passages. After 20 passages, passaged viruses (P20 and P15) and viruses without passage (P0) were treated with FBP or PBS with the indicated concentration of FBP for MDCK infection. At 18 h post-infection, viral loads in cell supernatants were measured by RT-qPCR to identify the sensitivity of passaged viruses to FBP.

### Haemagglutination inhibition assay

HA titres of H1N1 and FluB viruses were tested by TRBC. Viruses (8HA titre) were premixed with peptides (50 μg ml^−1^) or PBS for 1 h and then an equal volume of TRBC was added to the virus for incubation at room temperature for 30 min. The precipitates of TRBC were recorded for calculating the HAI activity. TRBC with untreated virus and neutralization antibody from serum served as a negative and positive control of haemagglutination inhibition.

### Antiviral analysis in vivo

BALB/c female mice (10–12 weeks for H1N1 virus and 10–12 months for SARS-CoV) were obtained from The University of Hong Kong Centre for Comparative Medicine Research, and female hamsters (4–8 weeks for SARS-CoV-2) were obtained from the Chinese University of Hong Kong Laboratory Animal Services Centre through The University of Hong Kong Centre for Comparative Medicine Research. Animals were kept in a biosafety level 2 laboratory (housing temperature ranging 22–25°C with dark/light cycle) and given access to standard pellet feed and water *ad libitum*. All experimental protocols followed the standard operating procedures of the approved biosafety level 2/3 animal facilities and were approved by the Committee on the Use of Live Animals in Teaching and Research of the University of Hong Kong [45]. To evaluate the therapeutic effect, mice were intranasally inoculated with 3 LD_50_ of A(H1N1) virus or hamsters were intranasally inoculated with 5000 PFU of HKU001a, 250 PFU of B.1.617.2 or 250 PFU of B.1.1. 529. For treatment, PBS, FBP (2 mg kg^−1^), zanamivir (1.6 mg kg^−1^), or chloroquine (2 mg kg^−1^) was intranasally inoculated to animals at 6–8 h after the viral inoculation. Two more doses were given to the challenged mice or hamsters the following day. Lung tissues were collected at day 2 post-infection. Survival and general conditions for the A(H1N1) challenge were monitored for 14 days or until death.

## Supplementary Material

Supplemental Material

## Data Availability

All data that support the study’s conclusions are available from the corresponding author upon request.
