# Supplementary material for: Fusion-inhibition peptide broadly inhibits influenza virus and SARS-CoV-2, including Delta and Omicron variants
Source: Emerg Microbes Infect. 2022 Mar 30;11(1):926–37. doi: 10.1080/22221751.2022.2051753 (PMC8973381; doi:10.1080/22221751.2022.2051753)
Supplement: Supplemental Material [file TEMI_A_2051753_SM8154.docx]

**Supplementary Information**

**Fusion-inhibition peptide broadly inhibits influenza virus and SARS-CoV-2 including Delta and Omicron variants**

Hanjun Zhao, Xinjie Meng, Zheng Peng, Hoiyan Lam, Chuyuan Zhang, Xinxin Zhou, Jasper Fuk-Woo Chan, Richard Yi Tsun Kao, Kelvin K. W. To, Kwok-Yung Yuen

**Fig. S1. Hemolysis assay of FBP in turkey red blood cells (TRBC).** TRBC were treated by the indicated concentration of FBP. Hemolysis (%) was normalized to TRBC treated by Triton X-100. Data are presented as mean ±SD of three independent biological samples.

**Fig. S2. FBP did not inhibit rhinovirus infection** (n=4). RD cells were pretreated with FBP (25 or 50 μg ml^-1^) for 1h (Pretreat cell) for viral infection, or virus was pretreated by FBP (Pretreat virus) for 1h for viral infection, or infected cells were treated by FBP at 1h post infection (Post infection). Viral RNA copies in cell lysates were measured at 16 hpi. There was no significant difference when compared with PBS. Data are presented as mean ±SD of four independent biological samples.













**P9RS**

**FBP**

**Triton**





**Fig. S3. FBP did not disrupt H1N1 viral particles.** A(H1N1) virus was pretreated by FBP (200 μg ml^-1^), P9RS (negative control peptide, 200 μg ml^-1^) or Triton X-100. Virus was negatively stained for TEM analysis. Red triangles indicated intact H1N1 particles. Scale bar =200 nm. Experiments were repeated twice.


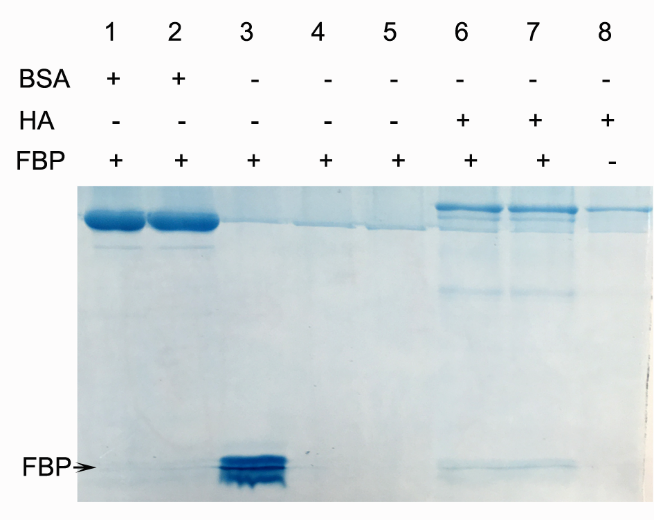


**Fig. S4. FBP binding to HA. FBP was premixed with BSA or HA for binding.** Lane 1-2, FBP mixed with BSA and then was filtered by 10kDa centrifugal filter to wash away the unbinding FBP. Lane 3, the positive control of FBP. Lane 4-5, FBP only was filtered by 10kDa centrifugal filter to wash away FBP. Lane 6-7, FBP mixed with HA and then was filtered by 10kDa centrifugal filter to wash away the unbound FBP. Lane 8, positive control of HA only. After washing the centrifugal filter, collect the proteins in centrifugal filter and proteins were detected by PAGE with Coomassie brilliant blue staining. Lane 6-7 indicated that FBP could bind to HA and was not filtered away by washing when compared with lane 1-2 and lane 4-5.


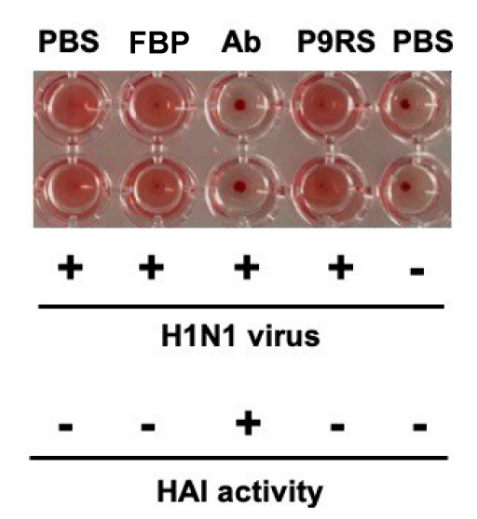


**Fig. S5. FBP did not show HAI activity on A(H1N1) virus.** A(H1N1) virus (8HA titer) viruses were pretreated by FBP (100 μg ml^-1^), P9RS (100 μg ml^-1^), neutralization antibody (Ab), or PBS. TRBC cells were added to treated or untreated viruses. Cell precipitates were recorded to show the HAI activity. Experiments were repeated twice.

**Fig. S6. FBP did not bind to viral surface to capture H7N9 virus.** FBP, P9R (positive control), P9RS and BSA (negative control) were coated on ELISA plate, which was blocked by BSA. A(H7N9) virus was added to ELISA plate for binding. After washing the unbound virus, viral RNA copies were measured to indicate the bound virus. Data are presented as mean ±SD of three independent biological samples.

**+pH5.0**

**+pH7.4**


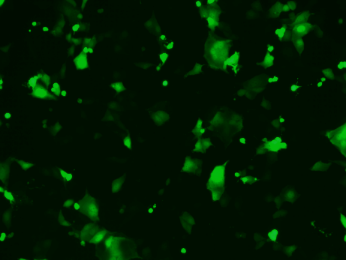

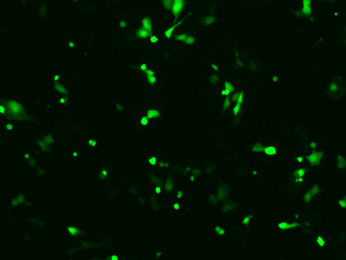

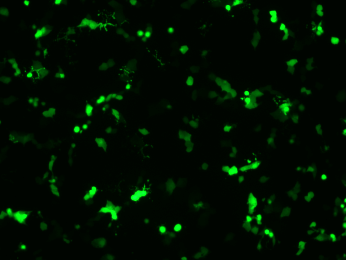

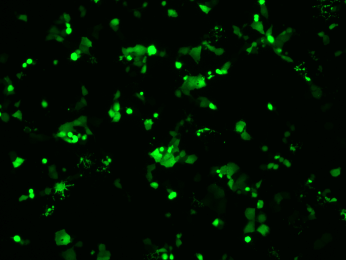


**+pH5.0+FBP-500**

**+pH5.0+FBP-125**

**+pH5.0+FBP-31**


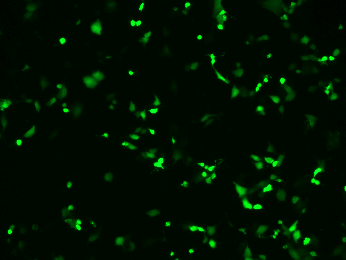


**Fig. S7. FBP could inhibit cell-cell fusion induced by FluB virus.** MDCK cells were transfected with pGFP and infected with 1MOI of FluB virus. At 18 hpi, infected cells were treated by FBP (500, 125, 31 μg ml^-1^) and then treated by pH5.0 or pH7.4. Infected cells treated by pH 7.4 were served as no fusion control and infected cells treated by pH5.0 were served as fusion control. Fusion pictures were taken at 4 h post pH treatment. The fusion cells (>50 μm) in pH5.0 treated cells and in pH5.0+FBP-31 treated cells were significantly bigger than the sizes (10-20 μm) of cells treated with pH7.0 and pH5.0+FBP-500. Scale bar=200 μm. Experiments were repeated twice.

**A**

**B**

**Fig. S8. FBP did not inhibit SARS-CoV-2 release and attachment. (A)** FBP did not inhibit SARS-CoV-2 release (n=4). VeroE6 cells were infected with SARS-CoV-2 virus. After 1 hpi, infectious media were removed and fresh media with FBP (25 μg ml^-1^) were added to infected VeroE6 cells for viral culture. Viral RNA copies in cell supernatants were measured at 8 hpi. (**B**) FBP did not inhibit SARS-CoV-2 attachment to cells (n=3). SARS-CoV-2 virus was treated by FBP (25 μg ml^-1^) or PBS for 45 min and then added to VeroE6 at 4 °C for 1h. Mock, no virus control. After washing, attached virus was measured by RT-qPCR. Data are present as mean ±SD of at least three independent biological samples.

**Fig. S9.** **FBP did not block spike binding to ACE2.** ACE2 was coated on ELISA plate. The binding of ACE2 with spike or spike pretreated by FBP, BSA or Ab was measured by OD_450_. Ab was the neutralizing antibody to block spike binding to ACE2. Mock, negative control without spike. ** indicates *P*<0.01 when compared with FBP. Data are presented as mean ±SD of four independent biological samples.

**Fig. S10.** U5 could block FBP binding to spike. FBP was coated on ELISA plate. The binding of spike to FBP was blocked by U5 when spike was pretreated by U5 (1.5 or 0.5, 0 µg ml^-1^). PA1 (1.5 µg ml^-1^) was the negative control peptide without blocking activity. Mock control without spike binding. ** indicates *P*<0.01 when compared with U5-0. Data are presented as mean ±SD of six independent biological samples.

**Fig. S11. FBP did not inhibit SARS-CoV-2 replication in Calu-3 cells.** SARS-CoV-2 (0.2 MOI) was treated by FBP (50 μg ml^-1^) or DMEM-F12 for 1h and then infected Calu-3 cells. Mock, no virus. Viral RNA copies in supernatants were measured by RT-qPCR at 30 hpi. Data are present as mean ±SD of four independent biological experiments.

**Table S1. Primers for RT-qPCR**

| Virus | Primer | Oligonucleotide sequence (5' to3') |
| --- | --- | --- |
| SARS-CoV-2 | S-F | CCTACTAAATTAAATGATCTCTGCTTTACT |
|  | S-R | CAAGCTATAACGCAGCCTGTA |
| H1N1 | M-F | CTTCTAACCGAGGTCGAAACG |
|  | M-R | GGC ATTTTGGACAAAKCGTCT A |
| H7N9 | M-F | CTTCTAACCGAGGTCGAAACG |
|  | M-R | GGC ATTTTGGACAAAKCGTCT A |
